# Unbiased Proteomics and Targeted Biomarkers Associated With Exceptional Longevity and Multimorbidity in Humans

**DOI:** 10.1093/geroni/igab046.1341

**Published:** 2021-12-17

**Authors:** Michelle Odden

**Affiliations:** Stanford University, Stanford, California, United States

## Abstract

Biomarkers ideal for geroscience trials could be those simultaneously identified using targeted and discovery assays and which strongly associate with complementary disease (multimorbidity) and longevity (exceptional survival) outcomes. To identify a tractable set of biomarkers for use in geroscience trials, we used the Cardiovascular Health Study (CHS), whose participant makeup closely aligns with the Targeting Aging with MEtformin (TAME) trial. In ~4800 CHS participants, quantitative assays of nine a priori-identified biomarkers were used to construct a biomarker index which strongly associated with the TAME primary outcome of mortality and multimorbidity over 6 and 10 years of follow-up. In ~3000 CHS participants, 1300 proteins were measured with unbiased aptamer proteomics and associated with survival to age 90 over 25 years of follow-up. Proteins in the biomarker index were identified as some of the strongest associated with survival to 90. This convergent evidence suggests these biomarkers may be well-suited for geroscience trials.

